# From the past to the present, optical coherence tomography in glaucoma: a practical guide to a common disease

**DOI:** 10.12688/f1000research.139975.2

**Published:** 2024-02-19

**Authors:** Izabela Zawadzka, Joanna Konopińska

**Affiliations:** 1 Department of Ophthalmology, Medical University of Bialystok, Bialystok, podlaskie, 15-081, Poland

**Keywords:** glaucoma, optical coherence tomography, retinal ganglion cells, open-angle glaucoma, optic neuropathy

## Abstract

Glaucoma comprises a group of disorders of the optic nerve that cause degenerative optic neuropathy, characterised by failure of neuroretinal rim tissue in the optic nerve head, retinal nerve fibre layer, and retinal ganglion cells. Glaucoma imposes a serious epidemiological threat, with an steady increase in the global number of cases. In the current ophthalmological practice, glaucoma is diagnosed via a series of examinations, including routine funduscopic examination, ocular tonometry, gonioscopy, measurement of the visual field, and assessment using the optical coherence tomography (OCT) technique. Nowadays, the OCT technique helps in systematising the diagnostic pathway and is a basic diagnostic tool for detection of early glaucomatous eye changes. It is also vital in assessing progression and monitoring treatment results of patients. The aim of this review was to present the OCT technique as a main tool in diagnosing and monitoring glaucoma.

## Introduction

Glaucoma is a chronic, multi-factorial, and progressive optic neuropathy characterised by loss of retinal ganglion cells (RGCs) and their axons, which causes characteristic optic disc and retinal nerve fibre layer (RNFL) structural changes. The RGCs transmit impulses received from photoreceptors through their axons to the optic nerve and then to the brain. The axons of RGCs pass through the collagenous structure lamina cribrosa (LA) and is covered with a myelin sheath that continues as the optic nerve. The main site of RGCs damage is LA, whose remodelling occurs as a result of an increased gradient across LA that causes papillary hypoperfusion, leading to impairment of the optic nerve fibres. These changes occur in cases of high intraocular pressure, low perfusion pressure, and/or low cerebrospinal fluid pressure, which leads to loss of RGCs. ^1^^–^^3^ Although glaucoma has a specific definition, there are cases with a different course like acute angle-closure glaucoma in which manifestation of the disease occurs rapidly, and well-controlled steroid-induced glaucoma that does not lead to the progression of glaucomatous neuropathy. ^4^^,^^5^ Assessment of structural defects of the optic nerve forms the basic principle for the diagnosis and monitoring of glaucoma. Introduction of new ocular imaging methods, particularly optical coherence tomography (OCT), has enabled the evaluation of optic nerves with the highest quality and objectivity. This enables early detection of glaucoma, which is critical to the treatment, and maintaining vision and good quality of life. ^3^

## Glaucoma classification

The group of disorders which forms the glaucoma disease spectrum has a complex aetiology, several risk factors, various manifestations, and several treatment options. Regarding the epidemiology in glaucomatous eye disease, we can distinguish two main types of the disease—the open-angle (OAG) and angle-closure (ACG) types—which may have primary and secondary causes. A sub-type known as normal tension glaucoma (NTG) also exists within the OAG type. ^1^^,^^2^^,^^6^

## Epidemiology

Glaucoma is the leading cause of blindness globally. In 2020, 3.6 million people suffered vision loss due to glaucoma, and 11% of all word blindness in adults over aged 50 years has been thought to be caused by glaucoma. In 2013, the prevalence of glaucoma was 2.93% among the population aged 40–80 years in Europe, and the prevalence of OAG is 2.51% in this age group. ^7^

In a recent meta-analysis, Tham *et al.* evaluated the number of patients with OAG and ACG globally and made a forward projection of the expected number of patients with glaucoma by 2040. They concluded that the number of glaucoma cases will grow rapidly, especially in Asian and African populations. The highest prevalence of primary ACG (PACG) is in Asia and that of primary OAG (POAG) is in Africa. This increase among Asian and African populations is possibly related to the increased life expectancy of the people, which will equal the life expectancy of European and North American populations in the near future. Another important factor proposed by the authors was that the African and Asian continents represent approximately more than 60% of the global population. The authors estimated that the number of people (aged 40–80 years) with glaucomatous eye disease in the world will increase from 64.3 million in 2013 to approximately 111.8 million in 2040. ^7^^–^^9^

In most countries, the increased incidence of glaucoma is associated with population aging. In addition, the higher identification of people with glaucoma is related with greater availability of diagnostic tests for glaucoma. Therefore, early detection of glaucoma is important to avoid the progression to advanced stages, and faster implementation of treatment to reduce the possibility of irreversible blindness. ^10^ OCT screening has high specificity and can be beneficial for the detection of glaucomatous eye damage in high risk populations; however, some authors state that this kind of screening may not be cost-effective. ^11^

## Risk factors

OAG risk factors can be divided into two groups: ophthalmological and systemic factors. When considering the ophthalmological risk factors in OAG, elevated intraocular pressure (IOP), high myopia, ocular perfusion pressure, central corneal thickness (CCT), and optic disc haemorrhages should be considered. ^12^ Notably, OAG risk increases at a higher IOP, but approximately half of all patients with OAG have IOP values in the normal range (10–20 mmHg). In contrast, glaucomatous optic neuropathy might not occur in patients with increased IOP. ^2^ In an Ocular Hypertension Treatment Study trial, Kass et al. ^13^ found that reduction of elevated IOP (21–32 mmHg) by 22.5% decreased the 5-year risk of developing OAG from 9.5% to 4.4%. The Ocular Hypertension Study proved that thinner CCT is a significant predictor of the development of OAG in patients with ocular hypertension, and that a patient with a CCT of ≥555 μm had a 3-fold higher risk of developing glaucoma within five years compared to a patient with a CCT >588 μm. ^14^ In addition, men have a higher risk of developing OAG compared to women. ^15^ Interestingly, an enlarged optic nerve disc in a myopic eye and increased thinning of the LA due to eye movement and increased shear forces in people with elongated axial length globes can also be significant factors that determine an increased risk of future glaucoma. ^1^^,^^15^

Moreover, systemic risk factors of OAG include African ethnicity, family history, smoking, age, genetic factors, as well as systemic hypertension (HT), nocturnal decrease in blood pressure, Raynaud syndrome, atherosclerosis, obesity, type 2 diabetes mellitus, and severe migraine headaches. ^12^ The prevalence of POAG in the European population increases with age, from 0.4% at the age of 40–44 years, to 2.7% at the age of 70–74 years, and up to 10.0% for persons over the age of 90 years. ^8^ HT is associated with a 16% increase in the risk of OAG, and the severity of glaucoma was found to be higher in patients with HT than that in individuals with normal blood pressure. ^16^^,^^17^ Conversely, hypotension may also be associated with the risk of glaucomatous optic neuropathy by reducing ocular perfusion pressure. ^18^^–^^20^ For PACG, ocular risk factors include swallowing of the anterior chamber, globe axial length shortening, increased lens thickness, and the anterior positioning of the lens. The demographic risk factors include increasing age, female sex, and East Asian ethnicity. ^21^

## Symptoms

ACG and OAG have no subjective symptoms, but in patients with ACG may exhibit a sudden manifestation due to acute primary angle closure that can cause ocular pain. less frequent visual impairment, conjunctival injection, rock-hard globe, and rare nausea and vomiting. It is essential for the ophthalmologist to diagnose patients accurately based on these symptoms and intervene with immediate treatment. Patients with OAG do not exhibit any symptoms for a long time as OAG develops slowly with a low intensity; hence, in most cases, it is unnoticed by the patient until it has reached an advanced or late stage. ^1^^,^^22^ In addition to the patient’s delayed awareness of visual field defects, the defects usually do not correlate in both eyes and leads to compensation due to binocular vision. ^1^

## Glaucoma detection

Glaucoma can be diagnosed using the following tests: funduscopic examination, ocular tonometry, gonioscopy, measurement of the visual field (VF), and assessment using the OCT technique. ^1^^,^^2^ The core of glaucoma diagnosis is the assessment of optic nerve changes mainly in funduscopic examinations, VF tests, and using OCT techniques.

### Fundoscopic Examination

The fundoscopic examination in the assessment of the optic nerve head (ONH) is based on the evaluation of the neuroretinal rim (NRR), which is an area between the optic disc margin (DM) and cup edge. ^23^ The characteristic ONH changes in glaucoma include a focal or diffuse NRR thinning, specifically in the superior or inferior quadrant of the optic disc with an enlargement of the ONH cup to disc ratio > 0.5. ^24^ The proposal by Jonas et al., ^25^ the inferior > superior > nasal > temporal (ISNT) rule, states that the thickness of the NRR decreases in the previously mentioned order, and that the NRR in glaucomatous optic discs disturb this relationship. However, several papers also deny the usefulness of ISNT assessment. ^26^

### Visual field

An important examination for detecting and monitoring functional impairment of the optic nerve in glaucoma is the assessment of VF using static automated perimetry (SAP). VF abnormalities are associated with loss of RGCs and their axons. The glaucomatous visual field defect can the diagnosed using the Hoddap–Parrish–Anderson criteria, ^24^ which include the following:

• Hemifield test that exceeds the normal limit on at least two fields.
• Typical of a glaucomatous changed eye cluster of three or more depressed non-edge points, with a P < 5%, and one that is depressed at a P < 1% on two consecutive fields.
• A corrected pattern standard deviation that occurs in less than 5% of normal fields on two consecutive fields.

Glaucoma diagnosis should be performed in the case of dissymmetry of VF between the eyes. The characteristic defects in VF in glaucoma mainly include narrowing of the peripheral VF, temporal island of VF, temporal wedge, Roenne’s nasal step, and different types of scotomas (paracentral, ring-shaped, Seidel’s, and Bjerrum’s). ^24^

VF examination is highly subjective depending on patient compliance and concentration. The quality of the exam is determined by different factors, such as the patient noncompliance and fatigue especially in elderly patients. In addition to artefacts, such as blepharoptosis, lens defects and refractive errors also decrease the quality of the exam. ^27^ The perimetric examination should be performed on the same device and with the same protocol (e.g., 10-2, 24-2, 30-3) at least three times a year in the first year after the diagnosis – this increases the reliability of the results. ^1^ The result maps out the patients’ VF, which can be helpful in assessing the baseline and tracking disease progression. In the case of a ganglion cell loss of less than 40–50%, there are no reliable VF defects in threshold perimetry, thus, the concept of preperimetric glaucoma was proposed as typical glaucomatous eye changes without the presence of VF defects detected by a perimetric examination. ^24^ Nowadays, the availability of a new technology, such as OCT-based fundus imaging, improves the assessment of preperimetic glaucoma progression. ^28^

### OCT

The OCT technique is superior because the automated perimetry examination can lack reproducibility and has some degree of non-objectiveness. Moreover, the OCT examination shows evidence of structural changes, including ONH damage and RNFL thinning, before functional loss is detected by standard automated perimetry. Furthermore, OCT is reliable when performed on both normal and glaucoma patients, and shows superiority in monitoring patients who are unable to undergo VF testing, including children, the elderly, and those with dementia. ^27^ In ophthalmology, the OCT technique is a basic tool in daily clinical practice. ^29^ Hence, its superior resolution renders it as a reliable tool for retinal morphology assessment and the measurement of retinal thickness, which have become important in evaluating patients with wet age-related macular degeneration. ^30^ Retinal thickening is also frequent in patients with type 1 or 2 diabetic retinopathy, approximately 15% of whom develop macular oedema, where OCT can also be one of the first-choice diagnostic tools. The third and leading reason for OCT evaluation is patients with glaucoma, which causes RNFL atrophy and visual loss in advanced stages, is that OCT evaluation can detect RNFL thinning at an early stage, often before a patient’s visual loss, leading to early-stage glaucoma detection. Additionally, OCT is used in nearly all fields of ophthalmological disorders, such as monitoring and follow-up for eye surgery, macular holes, vascular occlusions, and examination of pathologies in the anterior segment of the eye. ^31^

## Optical coherence tomography – history and basic principles

OCT is a diagnostic imaging method that produces a cross-sectional tomographic image of the eye structures with near-histologic and ultrahigh resolution. ^30^ It was originally developed by researchers from the Massachusetts Institute of Technology in the early 1990s, evolving from optical coherence domain reflectometry (OCDR), which is a method used for finding faults in fibre-optic cables and network components. The first OCT introduced was a two-dimensional, tomographic imaging modality for the biological system. OCT is a similar method to that of ultrasound, but it uses light instead of sound, and produces images of a higher magnitude (it can deliver images of 1–15 μm contrary to traditional ultrasound techniques which have significantly lesser spatial resolution). ^32^^–^^34^

OCT imaging is an important non-invasive technology in the detection and monitoring of glaucomatous structural damage. Over the years, OCT techniques have evolved from time-domain OCT (TD-OCT) to spectral-domain OCT (SD-OCT) and swept-source OCT (SS-OCT), which have improved the resolution and speed of scans. TD-OCT, a previously used technique, encoded the location reflections in time information and related the location of the reflection to the position of the moving reference mirror. Therefore, imaging of the fundus was possible, and changes in glaucoma could be evaluated and observed over time; however, the main disadvantage of this method was the slow scan time and two-dimensional imaging, which made the technique prone to artefacts. Another limitation of the TD-OCT technique was the limited maximal oscillating speed of the reference mirror, which performed only 400 axial scans. OCT technology has advanced since its first application in ophthalmology and continues to evolve rapidly. Hardware advances in commercial systems has improved resolution and increased scanning speeds, as a result of the replacement of the TD-OCT technique by the SD-OCT method. SD-OCT, also known as the Fourier-domain OCT (FD-OCT) method, allows acquisition of all information from a single axial sample simultaneously through the tissue by evaluating the frequency spectrum of the interference between the stationary reference mirror and reflected light. This not only improves scan density and resolution, but also significantly reduces imaging artefacts by reducing the scan acquisition time. ^3^^,^^35^ In addition, SD-OCT produces 3D datasets that can enable a greater assessment of the ONH parameters in the early evaluation of glaucoma and improved progression monitoring. From 3D images, we can acquire accurate information about the ONH parameters, including the disc area, rim area, cup-to-disc ratio, cup volume, and RNFL thickness. ^36^

SS-OCT is a variation of FD-OCT. The hardware in SS-OCT differs from that in SD-OCT, including the light source, bulk optics components, and photodetection device. In SS-OCT, the wavelength of the light source is approximately 1 μm, which sweeps across a narrow band of wavelengths, whilst the SD-OCT utilises a broadband light source. For the detection of the light waves, SS-OCT uses a point photodetector, whereas SD-OCT uses a spectrometer consisting of a diffraction grating, Fourier transform lens, and a detector array or linescan camera. Notably, the light source of the SS-OCT system is more complex; however, the design of the photodetector device is simpler, which gives the system faster scanning speed, resulting in higher scan rates with deeper tissue visualisation. ^37^^,^^38^

## Optical coherence tomography in glaucoma — diagnosis and progression

The introduction of the OCT technique over 20 years ago has allowed for supplemental, quantitative, and less operator-dependent precision with near histological-like spatial resolution assessment of glaucomatous eye changes. Using the OCT technique, we can acquire an automated segmentation of the retinal tissue layers, including the macula area, RFNL of the peripapillary region (pRNFL), and ONH area. ^3^ The macular area and pRFNL are the most important parameters in the assessment of glaucomatous eye changes. The OCT acquisition of the ONH region enables accurate and reproducible measurements of ONH parameters, such as the disc area, rim area, cup-to-disc ratio, cup volume, and RNFL thickness, but the large inter-individual variability reduces the reliability of ONH parameters. ^3^^,^^39^

### pRFNL area

**Figure 1.**
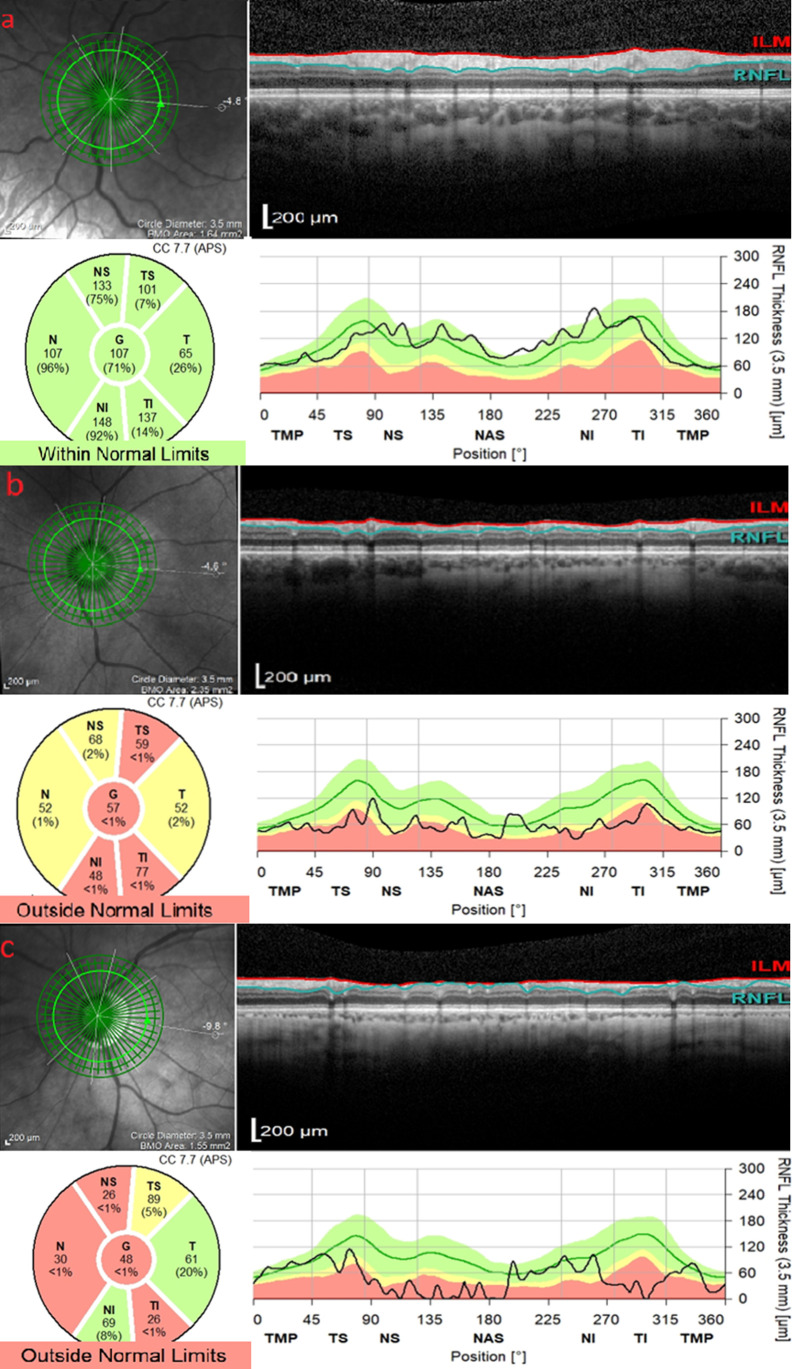
Analysis of peripapillary nerve fiber layer thickness; a) in a non-glaucomatous emmetropic eye; b) in a glaucomatous emmetropic eye; c) in a glaucomatous myopic eye. The retinal nerve fiber layer is situated between the internal limiting membrane (red line) and the boundary separating the retinal nerve fiber layer from the ganglion cell layer (green line). Images: Department of Ophthalmology, Medical University of Bialystok Clinical Hospital.

Thus far, the most popular OCT parameter in assessing glaucomatous eye changes is the pRFNL. pRNFL thinning in OCT is often one of the first signs of glaucoma, and it can be detected even before changes in the VF are observed. ^39^ In the diagnosis and assessment of glaucoma progression, the average pRNFL thickness, especially the pRNFL thickness in the inferior and superior quadrants, should be considered. Thinning of this parameter can have a significant impact on early glaucoma diagnosis, when the VF parameters are normal. The OCT analyses provide the RNFL thickness curve adjusted to the normative databases where green, yellow, and red colours are assigned to normal, borderline, and abnormal values, respectively (RNFL thickness values under the 99th percentile of normal database) ( Figure 1). Early-stage glaucoma known as ‘green disease’ occurs when green labelling of OCT parameters indicates normal values despite the presence of glaucoma or progressive glaucomatous damage. Thus, in some cases the RNFL parameter must be still monitored even when the curve is on the green area. One must especially consider the fact that an asymmetrical thinning, comparing both eyes, with a value greater than 9 μm in the average pRNFL thickness, can suggest early glaucomatous degeneration. ^40^^,^^41^ Some cases of reduced RNFL thickness with red labelling of OCT parameters, despite lacking glaucomatous changes, are considered as ‘red disease’, which can occur in cases of myopia. When monitoring for glaucomatous progression using OCT, the physician must be aware of the accompanying and the abovementioned age- and software-related artefacts and the floor effect. Age-related pRNFL thinning rate, which occurs in healthy eyes at a mean rate of −0.48 μm/year to −0.60 μm/year depending on the OCT device, must also be considered. Software-related artefacts also play a significant role in the accurate assessment of glaucomatous eye damage and/or progression; most of them are operator-dependent and include displacement of optic disk boundaries, myopic eyes, and/or significant media opacities, resulting in erroneous pRNFL values. ^42^ Glaucomatous progression must be suspected when there is a global decrease of ≥5 μm in the average pRNFL thickness. Another important factor of progression is a decrease of ≥7–8 μm in the sectoral pRNFL thickness. ‘Floor effect’ occurs when the pRNFL values decrease to a point where they are undetectable by OCT. Notably, the average pRNFL floor value ranges from 49.2 μm to 64.7 μm depending on the OCT device. This happens first in the pRFNL because the papillomacular region is more resistant to glaucomatous damage than the pRNFL; accordingly, measuring the thickness of the papillomacular structure is of great significance in the evaluation of glaucoma in its advanced stages. Thus, it is crucial to understand that the floor effect is achieved earlier within the pRNFL, and later in the macular region. In such cases, we should consider the use of macular OCT and VF 10-2 testing. ^43^ Despite the pRFNL being a useful and the most commonly used tool in diagnosing glaucomatous eye changes in cases of high myopia, small or tilted ONHs, and swelling of ONH, the measurement result can be unreliable; in these cases, the macular area parameters should be used as reference points. ^44^

### Macular area

Early-stage glaucoma can cause thinning of the macula area, which consists of macular RFNL (mRFNL) and ganglion cell layer with inner plexiform layer (GCIPL); GCIPL contains RGC bodies and RGC dendrites, which forms the ganglion cell complex (GCC = mRFNL + GCIPL). Assessing the macular region in this case is particularly important because it has the highest concentration of RGCs in the retina (approximately 50% of the RGCs of the entire retina). ^3^ During OCT evaluation of the macular area, the mRNFL thickness, GCC thickness, GCIPL minimum thickness, and GCIPL average thickness must be considered. When assessing these parameters, epidemiological factors, such as age and sex, must be considered. Additionally, central corneal thickness, axial length, and macular GCIPL attrition with aging, which occurs similarly to mRNFL at a rate of approximately −0.31 μm/year, are important and must be considered. In the assessment of the macular region, depending on the OCT device that is used, one can focus on assessing the GCC complex as a whole parameter or the GCIPL. The GCC thickness values range between 95.08 ± 7.88 μm in normal eyes, 83.30 ± 9.27 μm in early stage perimetric glaucomatous eyes, and 80.13 ± 9.60 μm in the moderate stage, and 75.08 ± 11.79 μm in the advanced stage. ^45^ When assessing glaucomatous eye changes, one must be aware that several studies suggest that in early glaucoma the GCC parameter is superior to the pRFNL, ^46^ but there is no clear consensus regarding this statement. ^47^ In establishing the diagnosis, it is crucial to inspect the average GCC thickness, as it is more reliable than the pRNFL in early stage glaucoma and preperimetric glaucoma. The average and inferior GCC thicknesses can be correlated with progressive visual field loss. The examination has greater diagnostic value with more advanced stages of the glaucomatous neuropathy when the signal strength values are higher. Moreover, it is unaffected by the increase in axial length, which results in more specific discrimination of glaucomatous changes in myopic eyes compared to that of RNFL thickness. ^46^ In advanced glaucoma, the early floor effect is achieved when assessing the pRNFL; thus, this parameter is excluded from OCT assessment in advanced stages. In such cases, one should consider a combination of OCT assessment of the macular region and VF 10-2 testing. The static automated perimetry offers various test protocols (10-2, 24-2, 30-2); the test points are localised approximately 6° from each other in the 24-2 and 30-2 protocols. ^48^^,^^49^ Due to the GCC thickness, which is strongly correlated with retinal sensitivity within a central range of 10° of the macula, the VF 10-2 should be considered as the main examination in determining the rate of progression in advanced glaucoma. Moreover, glaucomatous damage in advanced stages, when the RGC bodies can be displaced from their receptive fields of the macular area, can lead to false VF testing results. This can result in failure to assess the relationship between the defects and RGC damage; hence, in this case, a 10-2 test protocol with a 2° grid should be considered as the better choice as it displays more accurate results. ^50^ When assessing the GCIPL, Xu et al. ^51^ stated that the average GCIPL thickness is approximately 75.2 ± 6.8 μm in early glaucoma, which thins to 64.4 ± 8.4 μm in moderate glaucoma, and then to 55.6 ± 7.6 μm in advanced glaucoma. Furthermore, an average GCIPL thickness change of >4 μm is suggestive of glaucomatous progression. When monitoring progression, one must be aware that the macular thickness is visible as an arcuate defect on the thickness and progression-change maps. Although appearing later, an important parameter to consider is the floor effect, which is defined as a point at which no further structural loss can be detected. As Chhavi et al. stated, several studies have shown that in advanced stages of glaucomatous damage, when mRNFL thickness is <55 μm, the GCIPL thickness change still correlates with the damage measured using a 10-2 visual field; as mentioned above, the floor effect occurs when the average thickness of macular GCIPL is approximately 45 μm. ^39^ Several vendors provide different OCT devices, and not every OCT device can segment the GCIPL complex. Due to these limitations, some studies focus on assessing the GCC as a whole, whereas some assess the GCIPL complex as a solitary parameter. Despite this, the assessment of the GCIPL can be fraught with high error risk due to several coexisting factors, which warrants the consideration of the presence of an epiretinal membrane, areas of vitreous adhesion, myopic eyes, macular schisis, and drusen. ^52^^,^^53^

The above-mentioned groups of parameters comprise the basis of glaucomatous damage assessment. Importantly, in the clinical assessment of glaucoma progression, the expected results may vary due to a series of artefacts that can occur in the diagnostic process, such as the segmentation error artefact; decentration, especially in myopic eyes; and accompanying pathological changes in the eye, such as media opacities in the form of corneal haze, cataracts, vitreous debris, myelinated RNFL, epiretinal membrane, and swelling of the ONH; these artefacts may falsely decrease or increase RNFL measurements. ^39^

### Optical Coherence Tomography Angiography in Glaucoma

Although the complete understanding of glaucoma pathogenesis remains a challenge, a prevailing theory posits that vascular dysfunction within the ONH may indeed play a pivotal role in the development and progression of glaucoma. A noninvasive method, such as OCT-angiography (OCTA), enables the assessment of perfusion in targeted layers of the retina and optic nerve head, providing high resolution and replicability. OCTA techniques elevate flow signals through the assessment of reflectance signal amplitude across successive scans comparing the static to dynamic tissues. This enables the identification of dynamic structures, such as red blood cells within the vasculature, depicted as bright pixels, while regions of static reflectance, resembling constant tissue, are visualized as dark pixels. Therefore, unlike traditional fluorescein or indocyanine green angiography, OCTA examination allows for the visualization of retinal vasculature without the need for dye injection and facilitates the assessment of vascular networks across distinct retinal layers.

In glaucoma, the OCT angiography parameters predominantly utilized for evaluating ocular vascular flow are vessel density (VD) and flow index (FI). Decreased FI and VD in the vascular network of peripapillary retina and ONH have been observed in both preperimetric and perimetric POAG according to various studies. ^54^^–^^56^ VD is less affected by the floor effect in comparison with metrics assessing retinal thickness, making VD a valuable parameter in the advanced stages of glaucomatous progression.

Additionally, to that it is worth mentioning that some studies propose the possibility of identifying changes in OCTA metrics in specific sectors before deviations in RNFL thickness are identified.

The main limitations of OCTA include segmentation artifacts, typically associated with patient movement and a lack of fixation, reduced optical transparency, such as cataracts and vitreous floaters, and the absence of a normative database for clinical assessment and classification. Moreover, elevated intraocular pressure reduces the actual vascular density measurements.

However, more advanced OCTA technologies enable a reduction in the number of artifacts associated with patient non-compliance and improve the segmentation of retinal layers, providing a more accurate visualization of the vascular network. ^3^^,^^57^

OCTA is an immensely crucial diagnostic method. However, owing to its extensive range of applications, the continual development of new modalities within this technique, and the inherent challenges of the technology in glaucoma diagnosis, potentially resulting in varied diagnostic approaches, further comprehensive research in this field is necessary.

## Conclusion

Glaucoma is a progressive and degenerative disease that leads to irreversible vision loss. Through its rapid developments, which have led to increased resolution and scan speeds, the OCT technique has become a powerful tool for diagnosing and detecting glaucoma progression. All assessed parameters, including RNFL thickness, macular RNFL, and ONH parameters, are valuable tools for early assessment and monitoring of glaucomatous eye changes, and none of these parameters showed significant superiority over the others. Therefore, all parameters are valuable in assessing different stages of glaucomatous disease.

## Data Availability

Not applicable as this is a review article.
