# Supplementary material for: From the past to the present, optical coherence tomography in glaucoma: a practical guide to a common disease
Source: F1000Res. 2024 Feb 19;12:1186. Originally published 2023 Sep 22. [Version 2] doi: 10.12688/f1000research.139975.2 (PMC10951567; doi:10.12688/f1000research.139975.2)
Supplement: Peer review history

# Reviewer report: Reviewer response for version 2

**DOI:** 10.5256/f1000research.162403.r252316

**Published:** 2024-03-19

**Authors:** Marta Grannonico

**Affiliations:** 1 University of Virginia, Charlottesville, USA

The authors conducted a comprehensive analysis of the incidence, etiology and risk factors involved in the development of glaucoma disease. In this review they assessed all the diagnosis methods current available in the clinic to detect early sign of glaucoma. Specifically, they focused on the latest imaging technology, the optical coherence tomography (OCT). Overall, the review is well-written and informative. 

 To be more factual, I would suggest to add a citation in the introduction when referring to the RGCs receiving impulses that travel to the brain. And an additionally citation in the pRNFL area when referring to normal, borderline or abnormal values assigned to the RNFL thickness.

Is the review written in accessible language?

Yes

Are all factual statements correct and adequately supported by citations?

Partly

Are the conclusions drawn appropriate in the context of the current research literature?

Yes

Is the topic of the review discussed comprehensively in the context of the current literature?

Yes

Reviewer Expertise:

Application of optical coherence tomography (OCT) in glaucoma animal models and humans. Study of neurodegenerations in ocular diseases.

I confirm that I have read this submission and believe that I have an appropriate level of expertise to confirm that it is of an acceptable scientific standard.

# Reviewer report: Reviewer response for version 1

**DOI:** 10.5256/f1000research.153299.r220515

**Published:** 2023-11-30

**Authors:** Karanjit Kooner

**Affiliations:** 1 Department of Ophthalmology, University of Texas at Southwestern Medical Center, Dallas, TX, USA

An excellent review regarding the role of OCT in glaucoma, by Zwadzka and Konopinska, covers a wide range of clinically relevant information for ophthalmologists treating patients with glaucoma. The review combines the pathophysiology and epidemiology of glaucoma leading up to the benefits of relatively objective OCT technology. 

 The review however lacks examples of OCT scans in patients with glaucoma and in myopia. The authors should also touch on the role of OCT-angiography (OCTA) which provides details on the vascular parameters as well.

Is the review written in accessible language?

Yes

Are all factual statements correct and adequately supported by citations?

Yes

Are the conclusions drawn appropriate in the context of the current research literature?

Partly

Is the topic of the review discussed comprehensively in the context of the current literature?

Yes

Reviewer Expertise:

I am a glaucoma specialist practicing at a major academic center in Dallas Texas. USA.

I confirm that I have read this submission and believe that I have an appropriate level of expertise to confirm that it is of an acceptable scientific standard.
